# A New Deep Learning Based Multi-Spectral Image Fusion Method

**DOI:** 10.3390/e21060570

**Published:** 2019-06-05

**Authors:** Jingchun Piao, Yunfan Chen, Hyunchul Shin

**Affiliations:** Department of Electrical Engineering, Hanyang University, Ansan 15588, Korea

**Keywords:** image fusion, visible, infrared, convolutional neural network, Siamese network

## Abstract

In this paper, we present a new effective infrared (IR) and visible (VIS) image fusion method by using a deep neural network. In our method, a Siamese convolutional neural network (CNN) is applied to automatically generate a weight map which represents the saliency of each pixel for a pair of source images. A CNN plays a role in automatic encoding an image into a feature domain for classification. By applying the proposed method, the key problems in image fusion, which are the activity level measurement and fusion rule design, can be figured out in one shot. The fusion is carried out through the multi-scale image decomposition based on wavelet transform, and the reconstruction result is more perceptual to a human visual system. In addition, the visual qualitative effectiveness of the proposed fusion method is evaluated by comparing pedestrian detection results with other methods, by using the YOLOv3 object detector using a public benchmark dataset. The experimental results show that our proposed method showed competitive results in terms of both quantitative assessment and visual quality.

## 1. Introduction

Infrared (IR) and visual (VIS) image fusion technology is utilized to generate a composite image from multiple spectral source images for combining complementary information of the same scene. The input source images are captured from different imaging modalities with different parameter settings. The fused image is expected to be more suitable for human perception than any of the individual input image. Due to this advantage, image fusion techniques have wide applications in image processing and computer vision areas to improve the visual ability of human and machine vision. The general framework of image fusion is extracting representative salient features from source images of the same scene, and then the salient features are integrated into a single image by a proper fusion method. 

IR images are highly influenced by the external environment, such as light, fog, and smog. [1,2]. IR images are superior to VIS images in areas where the VIS image is invisible due to low-light conditions. [3,4]. Normal VIS imaging sensors capture the reflective properties of the objects, which can be edges and detail texture of objects. They are able to provide information for human visual perception. As stated above, due to differences in imaging mechanism, the intensities at the same pixel location in IR and VIS images often vary distinctly. A good IR and VIS image fusion method should be able to simultaneously keep the thermal radiation information in IR images and the texture detail information in VIS images.

In the last decade, many image processing methods have been proposed to extract salient features, such as multi-scale decomposition-based methods. In general, multi-scale decomposition consists of three steps, namely, decomposition, fusion, and reconstruction. Pyramids [5,6], wavelets [7,8,9], and shearlets [10,11,12] are the typical multi-scale transforms that are usually used in image fusion. Sparse coding is also a popular image encoding method, which has also been successfully applied to fuse multi-modality images [13,14,15]. With the prosperity of deep learning, using a convolutional neural network (CNN) or generative adversarial network (GAN) [16] has become a trend. In [16], a GAN-based method simultaneously keeps the radiation information from the IR images and the detail texture in VIS images. The drawback of this method is low computational efficiency. 

One of the most important problems in image fusion is to calculate a weighted map that incorporates information about pixel activity from different source images. In most existing image fusion methods, the goal is two-fold: namely, activity level measurement and weight allocation. In a traditional transform domain fusion method, the sum of the absolute values of the decomposed coefficients is used to measure activity level, and the “selected maximum” or “weighted average” rule is applied to other sources, depending on the measurement acquired. Distinctly, this kind of activity measurement and weight allocation is vulnerable to several factors, such as noise, distortion, and the intensity difference. Several activity level design and weight allocation methods have been proposed in recent articles [17,18] to improve convergence. However, it is not easy to design a feasible activity level measurement nor weight allocation strategy that can actually take into account all the key issues of convergence. Moreover, these two stages are designed individually without effective combinations in many fusion methods, which can significantly limit algorithm performance. 

In this paper, we address this problem from a different view point to overcome difficulties in (1) designing robust activity level measurement and (2) weight allocation strategies. Specifically, it trains a CNN [19], which encodes an image patch into a feature to map the source images directly to the weight map. CNN is a type of multi-layer neural network, which differs from the usual artificial neural network. It learns a layered feature representation for image data through multi-connected layers. Specifically, each layer contains a certain number of feature maps, which can be considered as the size of feature dimension in that layer. Each weight in a feature map is called a neuron. The operations, such as convolution, activation, and max-pooling, applied to neurons are used to connect multiple layers of feature maps [19].

To cope with two major difficulties in image fusion, we proposed a new effective deep learning based framework for CNN model training to combine the activity measurement and weight map generation for image fusion. The main contribution of this paper can be summarized as follows:

(1) We designed a CNN based learning scheme to measure the activity measurement and to generate the weight map automatically according to the saliency property of each pixel in the source image pair.
(2) The source image pairs were decomposed into low and high-frequency sub-bands by using a 3-level wavelet transform, and the fused image was obtained by reconstructing wavelet images with the scaled weight maps. It produced fewer undesirable artifacts for good consistency with human visual perception.
(3) We analyzed the experimental results systematically on both quantity and quality point of view. Quantitative assessment was carried out on twelve benchmark data, and the results were compared with those of eighteen representative prior art methods. In addition, the visual qualitative effectiveness of the proposed fusion method was evaluated by comparing pedestrian detection results after fusion by using YOLOv3 object detector on a public benchmark dataset.

## 2. Related Works

A review of recent IR and VIS image fusion techniques is summarized in [20]. Recently, image fusion technique has become a popular research field, and IR and VIS image fusion techniques are the crucial components. In terms of algorithms used, typically they can be classified in three general categories: pixel level, feature level, and decision level. 

First, the pixel-level based methods can be categorized as spatial domain-based methods and transform domain-based methods. Representative spatial domain-based algorithms are weighted average and block-based methods. The well-known transformation-based IR and VIS fusion algorithms are pyramid, contour, NSST, and other decomposition and reconstruction based methods. In addition to the methods mentioned above, there are various other methods of IR and VIS image fusion, such as sparse representation (SR), Markov random fields (MRFs), and principal component analysis-based method. Pixel-level based method is a trendy study for the whole image fusion area. 

Second, feature level-based methods rely on synthetic features and structural characteristics of images, such as edges, corner points, and textures, to segment the image or get a target distribution information from a local area of image. Then, information from the source images will be extracted and combined by applying certain fusion rules. The representative methods are based on object detection, edge extraction, saliency map extraction, and image segmentation. The feature level-based method requires a manual feature selection, as well as a manually designed fusion rule, and the fusion performance highly depends on the features and fusion rules. 

Third, decision level fusion is the most advanced option among the three levels, where a decision is made to integrate the targets based on a discriminative information according to a designed fusion rule. The fusion strategy is based on learning-based classifiers that generally quantify the reliability of classification. The shortcoming of decision level is the high dependency on detection for classification results.

The rest of this paper is organized as follows. The image fusion scheme based on automatic activity level measurement and weight map generation is introduced in Section 3. The performance evaluation and result analysis are discussed in Section 4. Finally, the conclusions are summarized in Section 5.

## 3. Fusion Scheme Based on Automatic Activity Level Measurement and Weight Map Generation 

**Figure 1:**
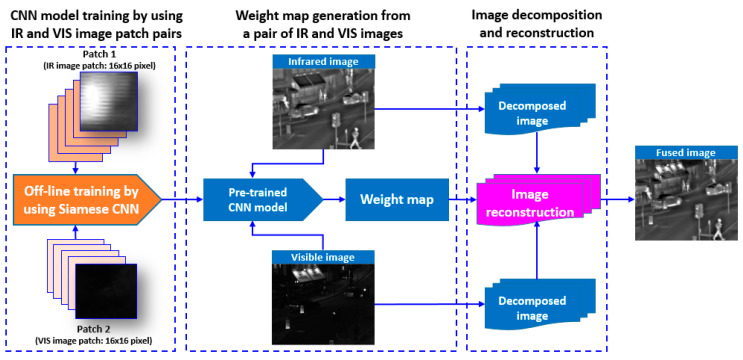
Block diagram of proposed visible (VIS) and infrared (IR) image fusion algorithm. CNN = convolutional neural network.

The aim of this work was to develop a CNN-based learning scheme to measure the activity level and to generate weight map automatically, according to saliency property of each pixel in the source images. In this work, we mainly focused on situations where the IR and VIS image pair was pre-registered. It can be seen from Figure 1 that the proposed method consists of three main steps: (1) CNN model generation by training a Siamese network, (2) weight map generation from a pair of IR and VIS images, and (3) image decomposition and image reconstruction. We designed a CNN-based training scheme to generate a two-class classification model that can compute the probability of each class. A large number of IR and VIS image patches with the size of 16 × 16 were used as training dataset. In weight map generation phase, the input were a pair of IR and VIS images, and weight map for the image pair was generated by using the trained CNN model. The weight map was the output of the training phase. The input image pairs were decomposed into low and high-frequency sub-bands, and weight maps were scaled to average the decomposed image pair. Lastly, the fused image was generated through weighted average and reconstruction.

### 3.1. CNN Design

**Figure 2:**
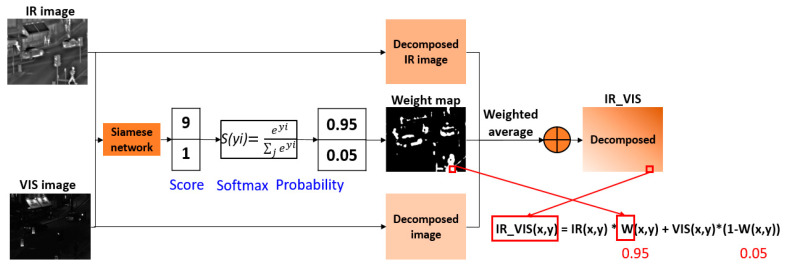
Schematic diagram of weight map generation and fusion scheme.

For this study, we regarded IR and VIS image fusion as a two-class classification task. The target is to generate a weight map whose value is ranging from 0 to 1 through training a CNN model. The coefficients in the weight map can be considered as the fusion rule that indicates the portion of each corresponding pixel intensity value in the source image during the weighted-average step. Figure 2 shows the weight map generation scheme of the proposed method. The input image pairs are encoded by Siamese network, and they are given a score which represents the saliency property of each source (VIS or IR). Consecutively, the probability calculated by using Softmax operation becomes the weight value in the weight map. The pixels with thermal radiation information in the IR image or the pixels belonging to detail texture in VIS image get higher probability. A weight map with the same size of the input image pair is calculated by using the pre-trained CNN model. In the weight map *W*, the brighter pixel indicates a value close to 1, and the darker is close to 0. For instance, if the value of a pixel(x, y) in the weight map is 0.95, then the weight of IR pixel is 95%, and the weight of VIS pixel is 5% at (x, y). The averaged pixel value is computed by IR_VIS(x,y) = IR(x,y) * W(x,y) + VIS(x,y) * (1-W(x,y)), where *IR_VIS(x, y), IR(x, y)*, *VIS(x, y),* and *W(x,y)* denote the pixel value of the weighted averaged image, IR image, VIS image, and the weight value at a certain position (x, y), respectively. 

**Figure 3:**
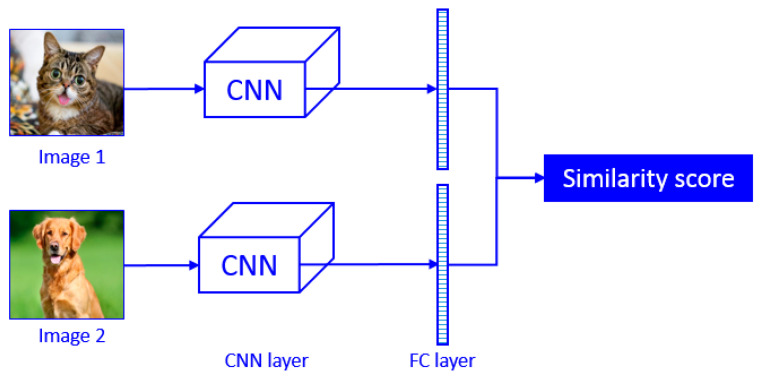
Basic concept of Siamese network.

A Siamese neural network was selected as the deep learning model in this work. Siamese neural networks are designed as twin networks, connected by their last layer by means of distance layer that is trained to predict whether two images belong to the same class or not. For instance, the two branches of CNN shown in Figure 3 are not different but are two copies of the same network. Therefore, they share the same parameters. Image 1 and image 2 are passed through the CNN to be encoded into a fixed-length of feature vector. If the two input images are from the same class, then their feature vectors must also be similar, while if the two input images are different, then their feature vectors will also be different. Thus, the element-wise absolute difference between the two flattened fully-connected feature vectors must be very different in the case of Figure 3. The fully connected layer of the two networks are then fed to a contrastive loss function based on the Euclidian distance, which calculates the similarity between two classes. Smaller Euclidian distance stands for higher similarity. This is the main concept of Siamese networks. 

**Figure 4:**
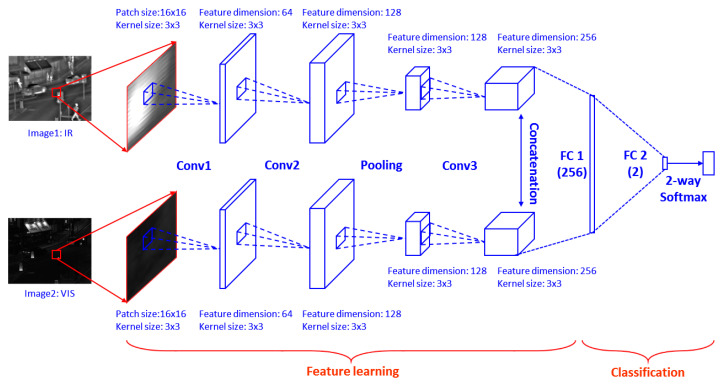
Architecture of Siamese network for patch-based feature extraction and training.

Figure 4 shows the CNN model used in the proposed fusion method. There are three convolutional layers and one max-pooling layer in each branch of the Siamese network. Table 1 shows specific parameter of the proposed CNN. Selecting image patch size is important. There is trade-off relationship between patch size and classification performance. A large patch size results in higher accuracy since more image features are encoded by neural network, but this increases the size of fully-connected layer significantly, which affects the efficiency. On the other hand, the training accuracy by using small patch size is not robust. By considering the above concerns and the size of the dataset image, we used 16 × 16 patches in this work. We concatenated the 256 feature maps obtained by each branch and fully-connected it with a 256-dimensional feature vector. Then a 2-dimensional vector is further fully connected with the first fully connected layer for Softmax operation. Lastly, the 2-dimensional vector is fed to a 2-way Softmax layer which generates a probability score of two classes. The full connection operation can be viewed as convolution with the kernel size that equals to the size of the input image. Assuming the size of the input image is h × w, then the size of the output weight map is [ceil(h/ 2) − 8 + 1] × [ceil(w/ 2) − 8 + 1], since the input image size is decimated to half after max-pooling operation, which is from 16 × 16 to 8 × 8. Conceptually, convolution, max-pooling, and concatenation play the role of feature extractor. Then, fully connected layers and the Softmax function classify the image patch pair with probabilistic values between 0 and 1. 

### 3.2. Training

The image patches for training are gathered from the TNO image fusion dataset and the OTBVS benchmark dataset. We made use of 2000 IR and VIS image pairs and divided them into small patches for training, instead of using whole images, as the input of CNN. We can use image of arbitrary size by doing so, and we extract image patches with stride of 2 pixels, for better efficiency, instead of doing it in a sliding window manner. Each training example is an image patch pair from source images. Let p1 be a patch from IR and p2 is the corresponding patch from VIS; then a training example {p1, p2} is defined as a positive example if its label is 1. On the contrary, the example is defined as a negative example if the label is 0. The training dataset consists of 400,000 positive samples and 400,000 negative samples. 

**Figure 5:**
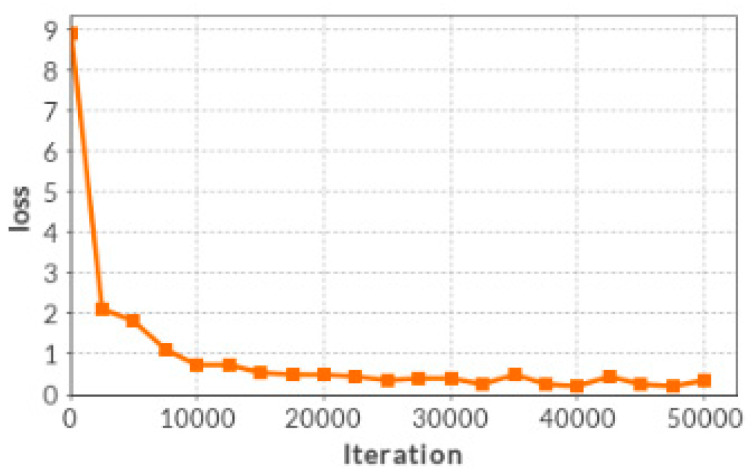
The loss reduction with the number of iteration.

The Softmax loss function is used as the objective of the proposed network. The stochastic gradient descent (SGD) is applied to minimize the loss function with iteration number of 50,000. The batch size is set to 128 for training. We trained our Siamese network on a popular Deep Learning Platform [21], which is based on the Caffe library. The initial weights of each convolutional layer are set by using the Xavier algorithm [22], which adaptively determines the scale of initialization according to the number of input and output neurons. The biases in each layer are initialized as 0. We set a same leaning rate of 0.0001 for all layers. We get a Siamese network model via loss function optimization after 50,000 iterations by using the 800,000 training examples. The model contains all weights and biases from each layer of network. The decreasing trend of the Softmax loss through iteration is shown in Figure 5. 

### 3.3. Final Weight Map Generation and Fusion Scheme

**Figure 6:**
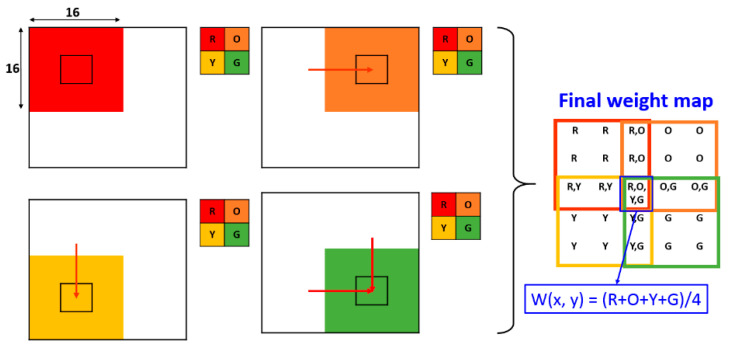
Mechanism for generating the weight map from input source images with the same size.

A CNN model is generated through training with a large number of IR and VIS image patch pairs. Since the output of CNN is a two-class probability distribution by using a Softmax classifier, a weight map *w* is obtained. In the training phase, since the kernel size and stride size of max-pooling is 2 × 2 and 2, the weight map size is reduced as mentioned in Section 3.1. By taking this into account, we know that every adjacent coefficient in *w* indicates the saliency property of an image path pair with size of 16 × 16. In order to make a weight map *W* that has the same size with source images, we redistributed the coefficients in *w* to a 16 × 16 patch with step size of 2 pixels, and took the average of overlapping patches. It can be considered as a reverse max pooling operation. Figure 6 shows an example of the weight map generation scheme with a weight map *w* of size 2 × 2. For instance, assume the weight map *w* consists of four pixels with values of R, O, Y, and G. Then, the final weight map *W* is obtained by assigning each pixel value in *w* to a 16 × 16 patch with stride of 2 pixels. Then, the pixel values of which multiple patches are overlapped can be calculated by averaging them. For example, in Figure 6, the value of the central pixels in *W* is (R + O + Y + G)/4. As mentioned in the Section 3.1, when the source images are of size h×w, the size of the output weight map is [ceil(h/ 2) − 8 + 1] × [ceil(w/ 2) − 8 + 1]. In reverse calculation, the size of the weight map should be [(ceil(h/2) − 8 + 1) × 2 + 14] × [(ceil(w/2) − 8 + 1) × 2 + 14], which is eventually equal to the size of the source image. 

**Figure 7:**
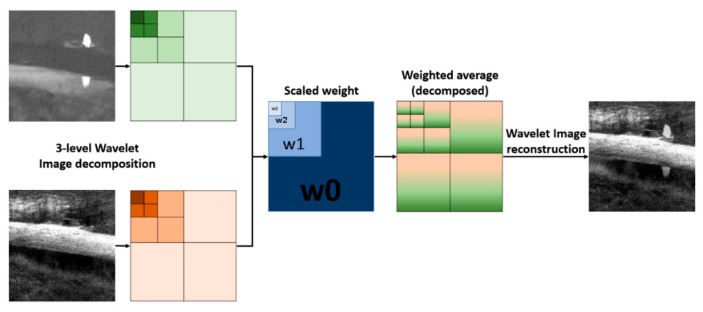
A 3-level wavelet transform-based image fusion scheme.

The IR and VIS images are captured by different imaging modalities, while the transform domain fusion method is suitable to produce less unexpected artifacts for good consistency with human visual perception. To cope with this issue, we decomposed both IR and VIS images by using a 3-level 2-D Haar wavelet transform [23], and the input image pairs were decomposed into low and high-frequency sub-bands. Since the size of the original image is down sampled during wavelet transform in each level, the weight map is scaled to match the size of down sampled images. Lastly, the fused image is obtained by reconstructing the 3-level wavelet images which cooperates with the weighted average by the scaled weight maps. The number of levels depends on the size of image to decompose. In this study, most of the images were sized (350–400) × (400–450) pixels. Images are down sampled and low-pass filtered in each level. If the number of levels is too large, images can be blurred due to the lack of high frequency component, which affects the reconstruction performance. The number of levels is selected by considering these factors. Details of wavelet transform-based image decomposition and reconstruction are introduced in [23]. The wavelet transform-based fusion scheme is illustrated in Figure 7. 

## 4. Experimental Results 

### 4.1. Benchmark Dataset and Experiment Environment

**Figure 8:**
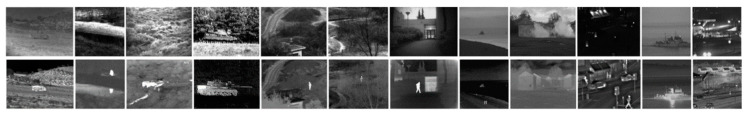
Twelve IR (**bottom row**) and VIS (**top row**) image pairs. From **left** to **right**: Athena, Bench, Bunker, Tank, Nato_camp, Sandpath, Kaptein, Kayak, Octec, Street, Steamboat, and Road.

To evaluate the performance of the proposed approach, we gathered images for training and fusion from TNO image fusion dataset and OTBVS benchmark dataset. The TNO Image Fusion Dataset contains multispectral imagery of different military relevant scenarios, registered with different multiband camera systems [24]. OTCBVS is a public available benchmark dataset for testing and evaluating novel and state-of-the-art computer vision algorithms [25]. Twelve test image pairs from the two image fusion datasets are shown in Figure 8. The VIS and IR images are strictly aligned to avoid the ghosting artifact in fused images. Furthermore, we used the Tokyo multi-spectral object detection dataset [26] to evaluate the effectiveness of the proposed method for pedestrian detection in low visibility circumstances.

We used a computer containing Intel i7 core CPU, 16GB RAM (Random Access Memory), under Linux operating system for CNN model training. An NVIDIA TITAN X GEFORCE GTX GPU (NVDIA, CA, USA) are used for accelerating the training process. The fusion experiment was carried out on a Windows system with an Intel i7 core CPU and 8GB memory with MATLAB implementation. For the objective performance evaluation, we ran the YOLOv3 object detector [27] on the same system with CNN training. 

### 4.2. Performance Assessment

Multi-spectral image fusion techniques have been extensively applied in a variety of areas, including object detection, target tracking, and surveillance. However, the practical applications heavily depend on the quality of the image fusion method. Therefore, the fusion performance should be evaluated in both a qualitative and quantitative manner [28]. Many assessment methods have been proposed to evaluate the performance of various IR and VIS image fusion methods and can be categorized as subjective and objective methods [29]. Subjective evaluation methods play an important role in evaluating the quality of fused images based on the visual perception. Subjective criteria includes image detail, object completeness, and image distortion. Nevertheless, the most straightforward subjective evaluation method is to apply a certain object detector on fused image, which as was carried out in this study. 

On the contrary, objective evaluation methods can quantitatively evaluate the performance of image fusion. They are very consistent with visual perception and are not easily biased by observers. A variety of objective methods based on fusion metrics have been proposed in recent years. They can be classified into information theory-based methods, image structure similarity-based methods, image feature-based methods, and human perception-based methods. Several representative image fusion method evaluation metrics are introduced and utilized in the experiment. Entropy (EN) and Mutual Information (MI) are the typical information-based methods. The EN of an image represents the amount of the information within an image on the basis of information theory [30]. MI measures the dependency between two images. More specifically, it quantifies the amount of information of source images is transferred to the fused image [31]. The structural similarity (SSIM) of image is a perceptual metric that quantifies the quality loss caused by processing [32]. Gradient information based metric Q^AB/F^ [30] quantifies the amount of edge information transferred from the source image to the fused image. Visual information fidelity (VIF) is a human perception-based metric [28], which addresses the notion of the image information extracted by the human visual system. For each of the assessment metrics above, a lager value indicates the better fusion result.

### 4.3. Results Analysis

We selected 18 representative prior art methods that were surveyed in the most recent paper [33] for comparing VIS and IR fusion performance with our proposed method. There were two main motivations for selecting prior art methods in this paper: (1) Prior art methods surveyed in the paper are representative, and the test codes of all prior art methods and evaluation metrics are available for performance evaluation. (2) The test images for performance evaluation and CPU time measurements are sufficient in terms of coverage and quantity. The typical methods surveyed in [32] are LP, Wavelet, NSCT3, dual-tree multi-resolution discrete cosine transform (DTMDCT), cross bilateral filter (CBF), hybrid multi-scale decomposition (HMSD), guided filtering-based fusion (GFF), anisotropic diffusion-based fusion (ADF), ASR, LP and SR (LPSR), orientation information-motivated PCNN (OI-PCNN), SF motivated PCNNs in NSCT domain (NSCT-SF-PCNN), directional discrete cosine transform and PCA (DDCTPCA), FPDE, two-scale image fusion based on visual saliency (TSIFVS), local edge-preserving LC (LEPLC), gradient transfer fusion (GTF), and IFEVIP. The LP, Wavelet, NSCT, DTMDCT, CBF, HMSD, GFF, and ADF are typical multi-scale transform-based methods, ASR and LPSR belongs to SR-based methods, OIPCNN and NSCT-SF-PCNN are typical neural network-based methods, DDCTPCA and FPDE are typical subspace based methods, TSIFVS and LEPLC are typical saliency-based methods, and GTF and IFEVIP belong to other method classes. 

We tested 18 reference methods and our proposed methods on 12 representative VIS and IR image pairs from the TNO dataset for qualitative and quantitative comparisons. Tested image pairs are exactly the same as the images tested in [31]. We used five typical assessment metrics, i.e., EN, MI, SSIM, QAB/F, and VIF, to evaluate the performances of different IR and VIS image fusion methods. For each of the assessment metrics, a lager value indicates the better fusion performance.

**Figure 9:**
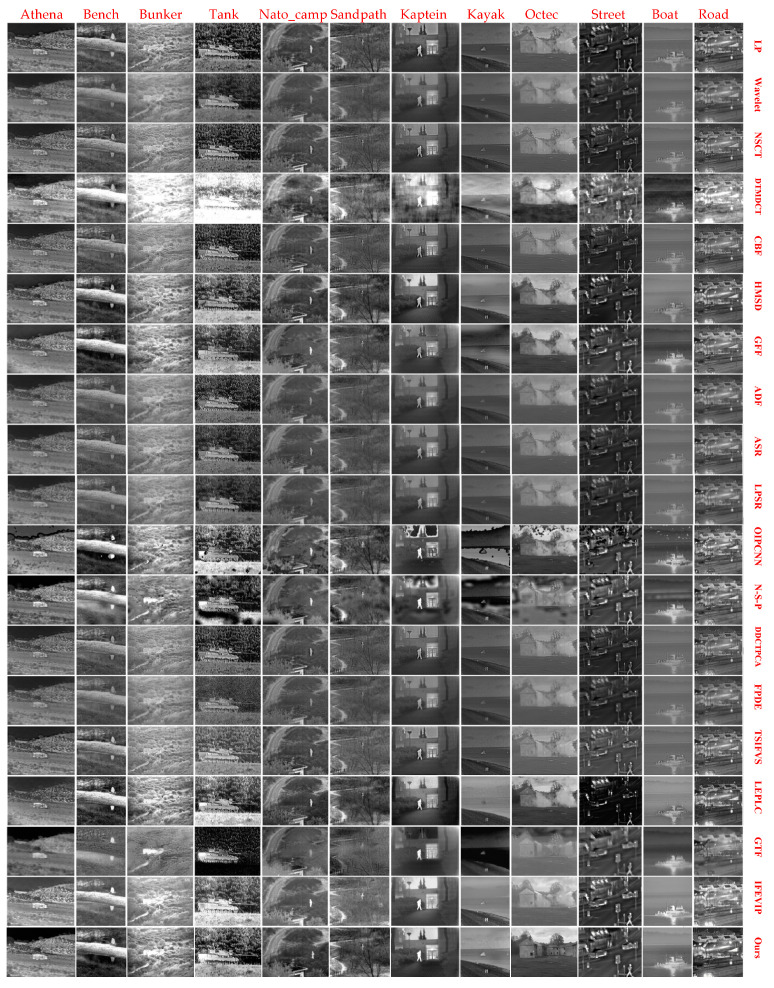
Qualitative performance comparison of 18 reference methods and proposed method on twelve IR and VIS image pairs. From **left** to **right**: Athena, Bench, Bunker, Tank, Nato_camp, Sandpath, Kaptein, Kayak, Octec, Street, Steamboat and Road. From **top** to **bottom**: LP, Wavelet, NSCT, dual-tree multi-resolution discrete cosine transform (DTMDCT), cross bilateral filter (CBF), hybrid multi-scale decomposition (HMSD), GFF, anisotropic diffusion-based fusion (ADF), ASR, LP and SR (LPSR), orientation information-motivated PCNN (OIPCNN), N-S-P, directional discrete cosine transform and PCA (DDCTPCA), FPDE, two-scale image fusion based on visual saliency (TSIFVS), local edge-preserving LC (LEPLC), gradient transfer fusion (GTF), IFEVIP, and ours.

We have tested and reviewed subjective visual quality comparisons of the 18 reference methods and proposed method on 12 IR VIS and IR image pairs. We evaluated qualitative performance according to the criterion of brightness preservation, artifact, and detail texture. Figure 9 shows the qualitative performance of 18 reference methods and the proposed method. The fusion results of DTMDCT are commonly brighter than other methods. The SR -based methods showed comparable results on both brightness preservation and artifact point of view. The results of neural network-based methods lack consistency with different test images. The sub-space-based methods and the saliency-based methods also produce comparable fusion results in terms of brightness and detail texture preservation. The proposed method exhibits excellent visual quality in preserving both thermal radiation intensity and detail textures, without bringing up unexpected artifacts. 

**Figure 10:**
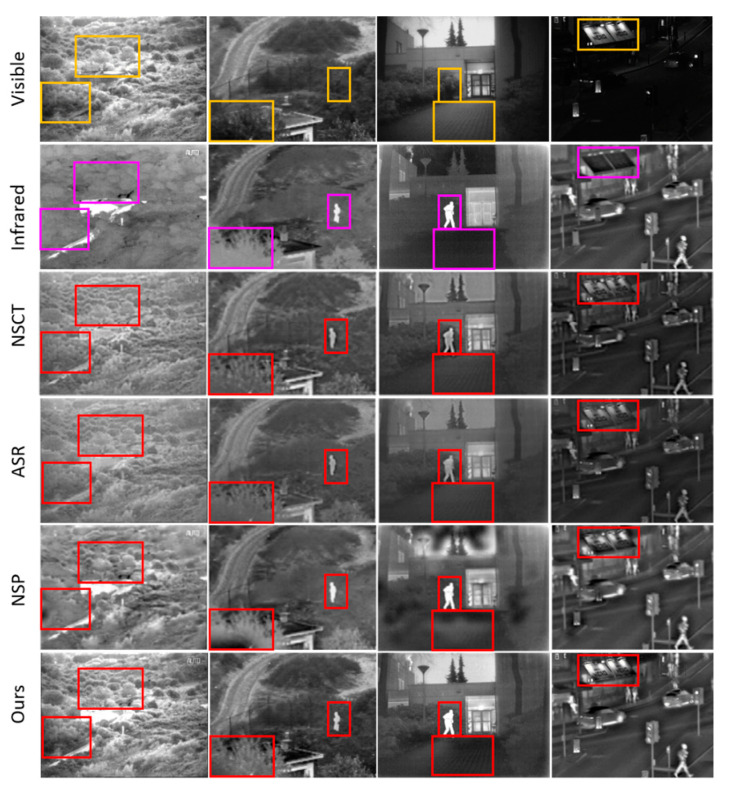
Detailed qualitative performance comparisons of three representative methods and proposed method, on four IR and VIS image pairs. From **left** to **right**: Bunker, Nato_camp, Kaptein, Street. From **top** to **bottom**: VIS, IR, NSCT, ASR, NSP, ours.

To further demonstrate the qualitative performance of the proposed method in the sense of detail texture and brightness preservation, we selected three typical methods to compare with ours, which are shown in Figure 10. Four sets of images, *Bunker*, *Nato_camp*, *Kaptein*, and *Street,* are selected from the benchmark dataset. NSCT is the representative multi scale transform-based method, ASR is the typical SR-based method, and NSCT-SF-PCNN is the representative neural network-based method. The sub-regions of images to compare are labeled by using yellow and magenta rectangles in VIS and IR images, respectively. The corresponding sub-regions are labeled by using red rectangle in fused images. (1) *Bunker*: In the proposed method, the detail textures and brightness from VIS image are well-preserved, which contrasts with the other three methods. (2) *Nato_camp* and *Kaptein*: In the proposed method, the thermal energy radiated from the human being is transferred more sufficiently from IR images to the fused image than other methods. The vertical yard pattern in the VIS image is also preserved in fused images. (3) *Street*: The brightness of the signboard is distinctly different in the proposed method and other methods. The overall performance of the proposed method shows promising visual quality on brightness and detail texture preservation point of view, without bringing up artifacts. 

**Figure 11:**
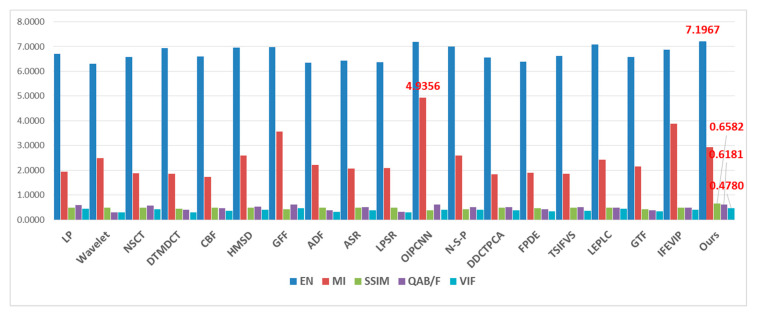
Tendency chart of average metrics value of 18 representative methods.

For objective quantity comparison, we reported the results of the five metrics using the 18 reference methods and our proposed method. Table 2 shows the metric values of 12 image pairs with proposed method, and the average value of respective metrics are compared in Table 3, in which the largest value in bold at each column indicates the best performance. For better observing the metrics value tendency, Table 3 is visualized as a stick chart in Figure 11. The OIPCNN and LEPLC methods show relatively high EN values, thus the fused image contains a large amount of information. The neural network-based methods achieve good values in MI, but poor values in SSIM. This result is coincident with distinct artifacts in the qualitative experiments. GFF and OIPCNN achieve good performance in Q^AB/F^, which indicates a relatively large amount of edge information is transferred from the source image to the fused image. The LEPLC and GFF methods exhibit good VIF values, which also matches with qualitative results. The proposed method produced the best results in EN, SSIM, Q^AB/F^, and VIF. For MI, the PCNN-based methods showed the best performance but exhibited low values in SSIM. 

**Figure 12:**
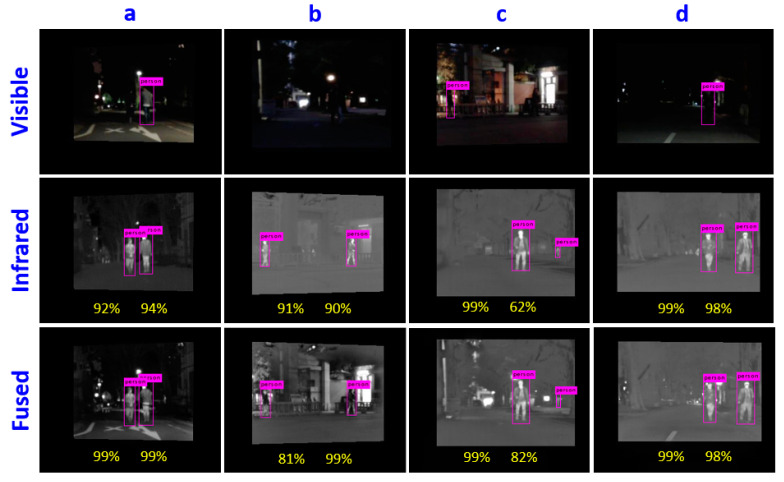
Pedestrian detection result comparisons between VIS, IR, and fused images from Tokyo multi-spectral object detection dataset. (**a**–**d**) are the image sets selected from the dataset.

In addition to the qualitative and quantitative performance comparisons, we tested the effectiveness of the proposed method by using night-time pedestrian detection. We applied a pedestrian detector integrated with the YOLOv3 on the VIS image, IR image, and fused image, respectively. The fused images are obtained by our proposed method. The pedestrians are missed due to low visibility in low-light or in night-time environments. By contrast, the missed pedestrians are detected in the IR image and the fused image, as shown in Figure 12. The percentage numbers marked in the images indicate the confidence value of the detection results, the higher the better. In most of the cases, the confidence values of pedestrian detection are higher in fused images than in IR images, except in the case of image b (81% vs 91%), in which the pedestrian area is overlapped with background objects. The proposed fusion method exhibits advantages on brightness and detail texture preservation, which affects the pedestrian detection performance.

The CPU timestamps were compared on the two sequences are shown in Table 4. The image size of the sequence is 270 × 360, and each value in the table indicates the average and standard deviation of the CPU timestamps of each method on two sequences. The results show that the efficiency of multi-scale transform-based methods are fast and stable. However, some approaches, such as ASR, NSCT_SF_PCNN, and DDCTPCA, are also relatively slow due to the complexity of algorithm. Our method takes around 19 s to process a pair of images. For real-time operation, the code transportation and parallel computing with hardware acceleration will be necessary, which remains a major part of future work. 

## 5. Conclusions

In this paper, we proposed a deep learning based IR and VIS image fusion method. In our method, a CNN-based image feature encoding and feature classification approach was applied to generate a weight map which indicated the probability of each source pixel to fuse from a pair of source images. By applying the proposed method, the key problems in image fusion, which are the activity level measurement and fusion rule design could be figured out at once. The visual quality of the method was proved by comparing performances using an object detector on a public benchmark dataset. The quantitative assessment results show that the CNN-based fusion method was more effective than manually designed methods in terms of noise, distortion, and the intensity difference. We believe that our method is very effective and robust fusion of pre-registered multi-spectral images. As future works, we intend to develop new deep neural networks for image fusion and to improve the efficiency of fusion procedure by implementing the algorithm with parallel computing units.

## Figures and Tables

**Table 1:** Parameter setting of CNN.

Layer	Patch Size	Kernel Size	Stride	Feature Dimension
Conv 1	16 × 16	3 × 3	1	64
Conv 2	16 × 16	3 × 3	1	128
Max-Pooling	8 × 8	3 × 3	2	128
Conv 3	8 × 8	3 × 3	1	256
Concatenation	8 × 8	N/A	N/A	512
FC 1	8 × 8	8 × 8	N/A	256
FC 2	8 × 8	8 × 8	N/A	2

**Table 2:** Metric values of proposed fusion method on 12 benchmark image pairs. EN = entropy; MI = mutual information; SSIM = structural similarity; VIF = visual information fidelity.

	EN	MI	SSIM	Q^AB/F^	VIF
Athena	7.2536	3.1623	0.6704	0.5797	0.8248
Bench	7.5477	4.4664	0.5558	0.7107	0.3162
Bunker	7.4693	3.2233	0.6282	0.6597	0.3683
Tank	7.7237	2.5543	0.4161	0.5280	0.2060
Nato_camp	7.1018	1.9957	0.7068	0.5042	0.4695
Sandpath	7.1106	2.5651	0.6540	0.5067	0.3771
Kaptein	7.1012	2.0924	0.7304	0.5565	0.4297
Kayak	6.9795	2.9931	0.6734	0.7590	0.5534
Octec	6.9670	4.2087	0.7733	0.7125	0.5512
Street	6.7090	2.6521	0.6409	0.6627	0.6720
Steamboat	6.9728	2.4326	0.8365	0.6042	0.3413
Road	7.4247	2.9362	0.6127	0.6338	0.6275
**Average**	7.1967	2.9402	0.6582	0.6181	0.4781

**Table 3:** Metric value comparisons of reference methods and proposed method on 12 benchmark image pairs.

	EN	MI	SSIM	Q^AB/F^	VIF
LP	6.7053	1.9353	0.4938	0.6011	0.4363
Wavelet	6.3003	2.4895	0.4869	0.2939	0.3028
NSCT	6.5850	1.8830	0.4945	0.5753	0.4213
DTMDCT	6.9425	1.8486	0.4431	0.3952	0.2956
CBF	6.5989	1.7220	0.4843	0.4752	0.3696
HMSD	6.9609	2.6005	0.4891	0.5284	0.3943
GFF	6.9890	3.5612	0.4344	0.6180	0.4681
ADF	6.3511	2.2094	0.4786	0.3823	0.3270
ASR	6.4384	2.0770	0.4898	0.5125	0.3767
LPSR	6.3580	2.0916	0.4856	0.3199	0.2910
OIPCNN	7.1803	**4.9356**	0.3906	0.6106	0.4069
N-S-P	6.9947	2.6022	0.4312	0.5015	0.4060
DDCTPCA	6.5567	1.8382	0.4851	0.5068	0.3927
FPDE	6.3974	1.9024	0.4617	0.4167	0.3338
TSIFVS	6.6270	1.8646	0.4898	0.5059	0.3632
LEPLC	7.0770	2.4172	0.4943	0.4810	0.4569
GTF	6.5819	2.1623	0.4236	0.3804	0.3440
IFEVIP	6.8685	3.8723	0.4865	0.4805	0.4061
**Ours**	**7.1967**	2.9402	**0.6582**	**0.6181**	**0.4780**

**Table 4:** CPU time comparisons of the proposed method and reference methods on benchmark sequences.

Method	*Nato_Camp*			*Duine*		
LP	0.004	±	0.0007	0.0044	±	0.0002
Wavelet	0.155	±	0.0382	0.1592	±	0.0018
NSCT	1.439	±	0.0092	1.4402	±	0.0096
DTMDCT	0.035	±	0.0018	0.0337	±	0.0019
CBF	6.143	±	0.0213	6.1211	±	0.0304
HMSD	0.544	±	0.0558	0.5492	±	0.0328
GFF	0.087	±	0.0067	0.0927	±	0.0091
ADF	0.177	±	0.0031	0.1730	±	0.0075
ASR	94.638	±	0.3782	94.6380	±	0.3199
LPSR	0.011	±	0.0026	0.0087	±	0.0005
OIPCNN	0.400	±	0.0021	0.3995	±	0.0018
N-S-P	72.047	±	0.2027	72.0280	±	0.1884
DDCTPCA	36.901	±	0.1771	37.1020	±	0.1162
FPDE	0.092	±	0.0040	0.0925	±	0.0043
TSIFVS	0.010	±	0.0019	0.0102	±	0.0014
LEPLC	0.149	±	0.0085	0.1575	±	0.0056
GTF	0.992	±	0.0609	1.3706	±	0.1052
IFEVIP	0.052	±	0.0012	0.0542	±	0.0015
**Proposed**	19.773	±	0.178	19.1633	±	0.132
